# The Inflammatory Milieu of Adamantinomatous Craniopharyngioma and Its Implications for Treatment

**DOI:** 10.3390/jcm9020519

**Published:** 2020-02-14

**Authors:** Ros Whelan, Eric Prince, Ahmed Gilani, Todd Hankinson

**Affiliations:** 1 Department of Neurosurgery, University of Colorado Hospital, Aurora, CO 80045, USA; eric.prince@cuanschutz.edu (E.P.); todd.hankinson@childrenscolorado.org (T.H.); 2 Department of Pediatric neurosurgery, Children’s Hospital Colorado, University of Colorado, Aurora, CO 80045, USA; 3 Morgan Adams Foundation Pediatric Brain Tumor Program, Aurora, CO 80045, USA; 4 Department of Neuropathology, University of Colorado Hospital, Aurora, CO 80045, USA; Ahmed.Gilani@childrenscolorado.org

**Keywords:** craniopharyngioma, inflammation, checkpoint inhibitors, Interleukin-6

## Abstract

Pediatric Adamantinomatous Craniopharyngiomas (ACPs) are histologically benign brain tumors that often follow an aggressive clinical course. Their suprasellar location leaves them in close proximity to critical neurological and vascular structures and often results in significant neuroendocrine morbidity. Current treatment paradigms, involving surgical resection and radiotherapy, confer significant morbidity to patients and there is an obvious need to discover effective and safe alternative treatments. Recent years have witnessed significant efforts to fully detail the genomic, transcriptomic and proteomic make-up of these tumors, in an attempt to identify potential therapeutic targets. These studies have resulted in ever mounting evidence that inflammatory processes and the immune response play a critical role in the pathogenesis of both the solid and cystic portion of ACPs. Several inflammatory and immune markers have been identified in both the cyst fluid and solid tumor tissue of ACP. Due to the existence of effective agents that target them, IL-6 and immune checkpoint inhibitors seem to present the most likely immediate candidates for clinical trials of targeted immune-related therapy in ACP. If effective, such agents may result in a paradigm shift in treatment that ultimately reduces morbidity and results in better outcomes for our patients.

## 1. Introduction

**Figure 1:**
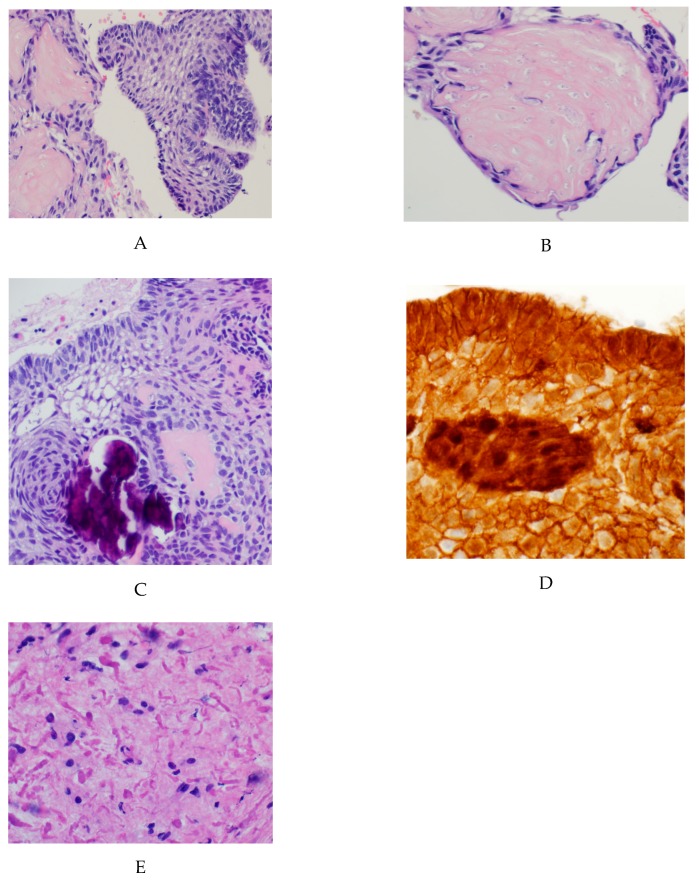
Classic histopathological findings in adamantinomatous craniopharyngioma: (**A**) H&E stained sections showing an epithelial tumor with palisading cells with aggregates of ‘wet’ keratin; (**B**) higher magnification view demonstrating keratinized ‘ghost cells’; (**C**) Higher magnification view demonstrating calcifications and stellate reticulum; (**D**) Immunohistochemical staining for Beta-catenin that is nuclear positive in a subset of tumor cells. (**E**) The surrounding brain parenchyma shows extensive gliosis with Rosenthal fibers.

**Figure 2:**
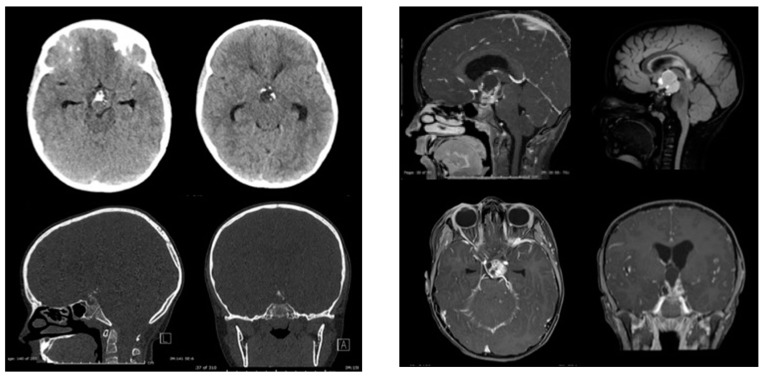
Computed Tomography (**Left**) andMagnetic resonance Imaging (**Right**) images from the same patient of a classic example of an adamantinomatous craniopharyngioma. This typical tumor is centered in the suprasellar region with mixed solid and cystic areas as well as areas of calcification as seen on the CT.

Pediatric Adamantinomatous Craniopharyngiomas (ACPs) are histologically benign brain tumors that often follow an aggressive clinical course. The tumors are most commonly centered in the suprasellar region and are believed to develop from remnants of Rathke’s pouch. Radiologically and grossly, these tumors appear as mixed solid and cystic lesions often with areas of calcification (Figure 1). Histologically, ACPs are heterogeneous tumors of epithelial origin [1,2]. The classic features consist of palisading epithelium, stellate cells, nodules of anuclear “ghost cells” and “wet keratin” as well as large areas of regressive changes (i.e., inflammation and calcifications, multinucleated giant cells, hemosiderin deposits, cholesterol clefts) [1] (Figure 2). Their proximity to critical neurological and vascular structures often confers significant neuroendocrine morbidity on patients [3]. Surgery remains the primary treatment strategy, but can result in significant morbidity, specifically damage to the hypothalamus, pituitary and optic apparatus, which results in long-term sequelae that can greatly impact a child’s quality of life [4,5,6]. In an era of personalized medicine and targeted therapies, ACP remains resistant to such advances. On the other hand, recent case reports of the response of papillary craniopharyngioma, a different tumor of the suprasellar region, to BRAF inhibitors have elucidated the great potential of targeted therapies in treating these tumors [7,8]. As a result, recent years have witnessed significant efforts to fully elucidate the genomic, transcriptomic and proteomic make-up of ACP in an attempt to identify potential therapeutic targets for the treatment of this disease [9,10,11,12,13,14,15].

### 1.1. The Central Role of WNT Pathway Overactivation in the Tumorigenesis of ACP

The one consistent genomic mutation that appears to be present in the majority, if not all, of ACPs is an activation mutation in the *CTNNB1* gene of the WNT/wingless pathway [9,11,16]. Most commonly this involves a point mutation in exon 3 of the *CTNNB1* gene. A number of studies have demonstrated various different mutations, most commonly involving serine or threonine phosphorylation sites encoded by exon 3 [13,17]. Ordinarily, and in the absence of WNT activation, beta-catenin is marked for destruction by a destruction complex consisting of AXIN, glycogen synthase kinase-3β (GSK3β), and APC, among other proteins. This complex binds to and phosphorylates specific residues encoded by exon 3 of *CTNNB1* and results in degradation of the protein [13,18]. In the presence of WNT activation, WNT ligands bind to Frizzled and its co-receptor LRP (Low-density lipoprotein receptor-related protein) at the cell membrane. This in turn leads to the activation of Disheveled (DVL) and the binding of AXIN at the cell membrane. Consequently, the normal destruction complex is broken up and beta-catenin is released. Eventually this stabilized beta-catenin will accumulate in first the cytoplasm, and subsequently the nucleus resulting in the expression of WNT pathway target genes [18]. In the pathological state present in ACP, the various point mutations prevent the binding of GSK3β to beta-catenin, and the subsequent phosphorylation of the serine and threonine residues. This results in a degradation-resistant form of beta-catenin, resulting in aberrant nuclear accumulation of the protein in certain cells within the tumor. In the nucleus, beta-catenin acts as a transcription factor, leading to overactivation of the WNT/beta-catenin pathway [16,18,19]. Although this aberrant overactivation of the WNT pathway is thought to be crucial in the pathogenesis of ACPs, the resulting nuclear accumulation of beta-catenin is only observed in a minority of cells, specifically in whorl like epithelial cell clusters (Figure 1D). These cells are thought to be crucial in the tumorigenesis of ACP and various mechanisms have been proposed as to how they may drive tumor growth [16,20,21] (Figure 2).

One such theory involves a paracrine mechanism whereby these cell clusters induce tumor growth by expressing a large array of growth factors, chemokines, and cytokines and act as a kind of signaling center that promotes tumor progression [21]. It has also been hypothesized that the nuclear accumulation of beta-catenin and overactivation of the WNT pathway in these cell clusters might also play a crucial role in the invasion of adjacent structures (e.g. hypothalamus and pituitary) in ACP [20]. Microscopically, a digitate invasion/growth pattern into structures such as the hypothalamus can be seen and is thought to be an important factor in the neuro-endocrine disorders frequently seen in children with ACP [3,22]. In addition, this invasive nature can preclude the neurosurgeon from obtaining a gross total resection at the time of surgery leading to tumor recurrence and a more aggressive clinical course. Hölsken et al. [20] noted that beta-catenin accumulating whorls/clusters are found at the tips of these invading projections of tumor and hypothesized that this may suggest a role for these clusters in the promotion of tumor invasion [20]. In addition, Apps et al. [23] used micro-CT to produce 3-D models of ACP tumor samples. Using this novel technique, they visualized cell clusters in tumor protrusions into surrounding tissue. In a separate paper, the same group used laser capture microdissection to separate out these cell clusters and analyze their transcriptomic profiles [10]. They found that these cell clusters express high levels of the FGF, BMP and WNT families of secreted factors and were able to demonstrate downstream activation of the MAPK/ERK that was particularly prominent at the tips of the invading tumor epithelium. These facts lend further credence to the theory that these clusters drive tumor invasion in a paracrine manner. Hölsken et al. [20] cultured a total of 6 ACP samples and measured their invasion capacity via two methods, namely Boyden chamber assays, and wound-healing assays. They then suppressed beta-catenin expression in the samples by introducing small interfering RNA (siRNA) directed against the *CTNNB1* gene and repeated the assays. They found that after treatment with the siRNA the accumulation of beta-catenin was significantly reduced and resulted in a significant decrease in tumor cell migration and invasion capacity [20]. They also demonstrated that the treatment with siRNA resulted in the reduced expression of the Fascin protein. Fascin is a member of the actin cross-linking family of proteins and plays a crucial role in cell-matrix adhesion, cell migration, and remodeling of the cell cytoskeleton/architecture [24,25]. In addition, the aberrant overexpression of the Fascin protein has been demonstrated in a number of cancers, including oral squamous cell carcinoma, and prostate cancer [24,26]. Hölsken et al. [20] demonstrated that the beta-catenin accumulating cells in ACP also over expressed Fascin. They then showed that treatment with the siRNA lead to a decrease in not only beta-catenin accumulation, but also Fascin levels. They proposed that this increase in Fascin expression may represent the mechanism by which WNT overactivation in these ACP cells may increase tumor cell migration and invasion into adjacent structures [20].

Given the seemingly crucial role of WNT overactivation in ACPs, targeting the WNT pathway would appear to represent an attractive strategy for tackling these tumors. The WNT pathway has been shown to play a crucial role in a number of cancers such as colorectal cancers, non-small cell lung cancer, and chronic myeloid leukemia [27,28]. This has resulted in significant efforts to better understand the pathway and to develop therapies that target it [27,28]. Despite all these efforts, no drug targeting the WNT pathway has been approved. The reasons for the difficulty in targeting the WNT pathway are legion and complex but one major area of concern is the important role the pathway plays in the maintenance of normal stem cells for tissue regeneration [27,29]. The potential issues that may arise with WNT pathway targeting was illustrated by Zhong et al. [30] who demonstrated significant intestinal toxicity associated with tankyrase inhibitors in mice.

Due to the difficulties that have been encountered in targeting the WNT pathway in more aggressive cancers, it seems likely that such therapies with acceptable efficacy and toxicity will remain elusive for some time to come [28]. It is unlikely such a therapy will become a viable option in the treatment of ACP in the near future and as a result, the need to discover other effective therapies has become imperative. ACP is a very rare disease and developing novel therapies specifically for this tumor type is currently not practical or realistic. As a result, much work has focused on identifying alternative targets with extant treatments, which may offer better results in the treatment of ACP. These efforts have resulted in the identification of multiple molecular pathways involved in the pathogenesis of ACP [6]. A number of these pathways result in the upregulation of pro-inflammatory/immune genes that may be amenable to targeted therapies [10,11,31,32,33]. The immune/inflammatory cells seen in ACP samples are varied and can include CD4-T-Lymphocytes, CD20-B-Lymphocytes, CD-68-Macrophages, and CD-56-NK cells. The presence of all these cells is not consistent among all ACP samples and this fact is reflective of the histologically heterogeneous nature of these tumors [34]. Work is ongoing to investigate whether these pathways may present potential therapeutic targets and ultimately leads to better outcomes and reduced morbidity for patients. The following is a review of the evidence that highlights the potential importance of the inflammatory/immune response in the generation of these tumors and the potential in targeting these pathways in the treatment of this often-devastating disease.

### 1.2. The role of the Inflammatory Response in Generating the Cystic Compartment in ACP

**Figure 3:**
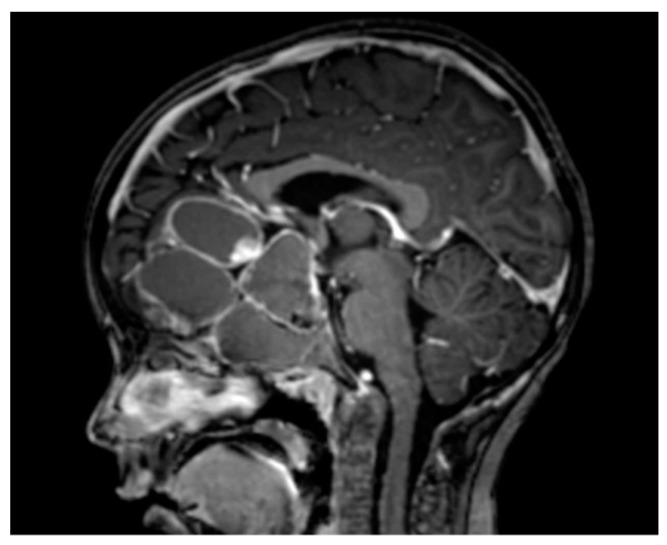
Example of an adamantinomatous craniopharyngioma with a massive cystic component.

ACPs often have large cystic components that contribute to the adverse clinical outcomes associated with the disease (Figure 3). Their large size and at times rapid growth can injure or exert mass effect on critical adjacent structures, such as the pituitary, the hypothalamus, the optic apparatus and third ventricle, which may necessitate urgent surgical intervention to preserve function and prevent morbidity and mortality. As a result, a better understanding of the pathogenesis of ACP cysts and the development of better treatments to limit their growth is clearly desirable. Numerous studies have analyzed the content of these cysts and the results of these studies have demonstrated a significant inflammatory content within them. A summary of some of these papers is presented in Table 1.

Some of the first work examining the role of inflammation in ACP pathogenesis was carried out by Mori et al. who demonstrated highly elevated levels of IL-6 in the cyst fluid of 15 pediatric ACPs and posited that IL-6 plays an important role in the inflammatory reaction associated with ACPs [37]. Another study that demonstrated the role played by the inflammatory response in the generation of the ACP cyst was that by Pettorini et al. [36]. Using high-performance liquid chromatography and mass spectrometry to analyze cyst fluid from 6 patients, they found high levels of alpha defensins 1–3, proteins that are present in neutrophils and are involved in the inflammatory-mediated response. Furthermore, their group demonstrated that these levels were significantly reduced after treatment with intracystic interferon alpha (IFN-alpha). They posited that the detection of these proteins suggested that the innate immune response was playing a critical role in cyst generation and that a possible mechanism of action of IFN-alpha in treating the cyst was via an immune-modulatory effect [36]. A later study by the same group performed more extensive proteomic analysis on nineteen patient samples [15]. In this study, they used reverse phase liquid chromatography in conjunction with high resolution ESI-I TQ-Orbitrap mass spectrometry to analyze ACP cyst fluid from nineteen children. In addition to again revealing elevated levels of alpha-defensins (that again were reduced after treatment with IFN-alpha), they also demonstrated elevated levels of several other proteins of inflammation. Specifically, these included alpha2-HS-glycoprotein, alpha1-antichymotrypsin and apolipoproteins.

In another study, Donson et al. [31] used cytometric bead analysis to measure the concentration of 24 cytokines and 11 chemokines in cyst fluid from five pediatric ACPs and five pediatric pilocytic astrocytomas (PAs). Their analysis demonstrated that six cytokines were present at statistically significant increased levels in ACPs versus PAs. These cytokines included IL-6, IL-10, CXCL8 (IL-8), and CXCL1 (GRO). Of these, levels of IL-6 demonstrated the greatest difference between ACPs and PAs. Apps et al. also demonstrated similar findings when analyzing the protein content of ACP cyst fluid [10]. They analyzed the content of cyst fluid from 6 patients with ACP using multiplex ELISA (Enzyme-linked immunsorbent assay). They found that the cyst fluid contained several proteins associated with inflammation, such as apolipoproteins, complement system proteins and immunoglobulins. In addition, their analysis revealed the presence of cytokines such as IL-1B, IL-6, IL-8, IL-10, IL-18 as well as TNF (Tumor necrosis factor) and Interferon gamma.

Further evidence for the role of inflammation in the genesis of the cystic component in ACPs is provided by the efficacy of treatment with IFN-alpha. IFN-alpha has been used with varying degrees of success in the treatment of multiple cancers [38]. The mechanism of action in the treatment of neoplasms is complex and multifaceted but likely involves the stimulation of an anti-cancer immune response [38]. The use of intracystic IFN-alpha in the treatment of cystic ACP has been established for several years and numerous studies have demonstrated its safety and efficacy [35,39,40]. The treatment involves the surgical placement of a catheter within the cyst with the position of the catheter confirmed radiologically prior to the administration of the drug. This method of treatment has been shown to delay disease progression and can allow the clinical team to delay a more definitive treatment via surgical resection and radiotherapy [35]. Such a delay is often desirable, as it by may allow a child’s developing brain to mature further prior to undergoing inherently risky surgery and radiation therapy. The mechanism of action of intracystic IFN-alpha in treating ACP has not been confirmed but, as in other cancers, is likely to involve an immunomodulatory effect. Indeed, the previously mentioned proteomic analyses would seem to lend significant weight to this argument [36].

### 1.3. The Solid Component of ACP Also Demonstrates Elevated Levels of Several Inflammatory Markers

Multiple studies have also identified high levels of cytokines and inflammatory markers in the solid component of ACPs, lending further support to the theory that inflammation plays a critical role in pathogenesis [18,31]. Gump et al. used micro-array data to demonstrate elevated levels of IL-6R in ACP relative to other pediatric brain tumors [11]. Subsequently, Donson et al. [31], utilized detailed transcriptomic analysis to demonstrate increased expression of pro-inflammatory mediators in ACP solid tumor tissue including IL-6, CXCL1, CLCL8, CXCR2, IL-10 and IDO-1. Separate work by Martelli et al. [32] used advanced proteomics to investigate the protein signature in ACP. In addition to identifying beta-catenin and its related proteins in solid tumor tissue from seven patients, their analysis also identified the presence of increased levels of alpha-defensins 1–4. As previously stated, these proteins are neutrophil-derived proteins that play an important role in the innate immune response and in inflammation. Their detection in the solid portion of ACP again seems to confirm that the inflammatory response plays an important role in ACP tumorigenesis.

A recent paper by Apps et al. used transcriptome analysis of tumor tissue from 18 patients to identify a pattern of elevated expression of several immune cell markers and immune system genes in ACP [10]. Furthermore, their analysis used immunohistochemistry to reveal the presence of both myeloid-derived and lymphoid-derived cells infiltrating both the reactive glial and tumor epithelial compartments in the ACP samples. They also found that multiple cytokine encoding genes were highly upregulated in ACP and that the expression of such genes correlated with the immune infiltrate and inflammatory cell markers. This would suggest that this upregulated cytokine expression is mostly derived from the infiltrating immune cells rather than from the tumor cells that are over expressing beta-catenin. Finally, they also utilized multiplex ELISA to analyze protein lysates from eight patient ACP samples and this analysis revealed the expression of IL-1B, Il-6, IL-8, IL-10, IL-18, and TNF-alpha in all the samples.

### 1.4. Immune Checkpoint Inhibitors and Their Potential Use in ACP

Immune checkpoint inhibitors have shown promise in the treatment of a number of cancers. Specifically, inhibition of the programmed cell death protein (PD1) and its ligand (PD-L1) with the agents, nivolumab and pembrolizumab, has resulted in improved survival in cancers including melanoma and non-small cell lung adenocarcinoma [41,42]. The availability of these agents and their relatively favorable side effect profile has resulted in numerous studies investigating their efficacy in various cancers/tumor types.

PD-1 is an important protein involved in inhibitory immune signaling and is an essential regulator of the adaptive immune response [43]. In cancers, PD-1-expressing tumor-infiltrating T cells can be disabled by PD-L1 expressed on the surfaces of tumor cells themselves or alternatively by PD-L1 on the surface of other infiltrating immune cells. The binding of PD-1 to its ligand results in the suppression of the immune response to the cancer cells [43,44]. Checkpoint Inhibitors reverse this process and allow T cells to once again attack the cancer. Predicting the response of a particular tumor or cancer to PD1 inhibitors such as nivolumab and pembrolizumab is difficult and Taube et al. aimed to identify those factors that best predicted a robust and meaningful response to therapy [44]. In a prior study by the same group they found that anti-PD-1 therapy produced an objective response in 20–25% of patients with treatment-resistant NSCLC, renal cell carcinoma and melanoma and that PD-L1 expression by tumor cells seemed to be associated with a response to therapy [45]. In their follow up study they aimed to further investigate various factors that might predict a response to anti-PD-1 therapy including PD-L1 expression by tumor cells, PD-L1 expression by infiltrating immune cells, PD-L2 expression by tumor cells and other tumor microenvironment factors. They found that in their cohort only the expression of PD-L1 by tumor cells correlated with both an objective response (as defined by the “Response evaluation criteria in Solid Tumors” or RECIST criteria) and clinical benefit (*p* = 0.025 and 0.005 respectively). The correlation of the expression of PD-L1 by infiltrating immune cells with a clinical response did not reach statistical significance although the correlation with clinical benefit was statistically significant (*p* = 0.038). Of note, expression of the PD-1 receptor on tumor infiltrating lymphocytes (TILs), expression of PD-L2 by tumor cells or TILs, and other microenvironment immune factors did not correlate with outcomes. In addition, it is important to reiterate that even in those tumors expressing PD-L1 on tumor cells, only 39% of patients (9 out of 23) had an objective response [44]. That being said, these therapies have provided an option for patients with aggressive and treatment-resistant cancers for whom previously there were few if any good options. Due to the efficacy of immune checkpoint inhibitors more and more work is being undertaken to identify other cancers and tumors that may be amenable to such therapy including craniopharyngiomas.

Recent work by Coy et al. [33] demonstrated the expression of PD-L1 in epithelial cells lining the cysts and intrinsic PD-1 expression in the beta-catenin over expressing whorl-like epithelial cell clusters in ACP. As previously discussed, these clusters are thought to play a pivotal role in tumor growth in ACP via a number of mechanisms [13,20,21], rendering targeting of PD-1 as an attractive potential therapy. Another study by Witt et al. [46] also demonstrated elevated PD-L1 expression in ACP. As mentioned above, numerous previous studies on other solid cancers have demonstrated that the expression of PD-L1 can be predictive of the response to the PD-1/PD-L1 inhibitors [44,45]. Again, as previously mentioned, such a finding far from guarantees a response and in these landmark papers they found that even in patients that expressed PD-L1 on tumor cells, the response rate to the treatment was only 39% [44]. In addition, Witt et al. [46] nicely demonstrated, using T cell exhaustion testing of various types of ependymomas, that elevated PD-L1 expression in tumors can be indicative of either tumor adaptations to hide from the innate immune response or due to normal T-cell antigen-activation, a known function of PD-1. In their study they utilized functional T cell exhaustion assays that stimulate T cells via exposure to Phorbol 12-myristate 13-acetate (PMA)/ionomycin. Subsequent to stimulations their group used a Milliplex Map Kit (Millipore) to measure the concentration of several cytokines including IFN-gamma. They found that infiltrating T-cells in RELA fusion supratentorial ependymoma did not secrete IFN-gamma. They concluded that this suggested that in the case of RELA fusion ependymoma, the increased expression of PD-1/PD-L1 results in the exhaustion of infiltrating T-cells and immune evasion by the tumor [46]. On the contrary, they found that in group B ependymomas (which also express high levels of PD-1), infiltrating T-cells were, in fact, capable of secreting IFN-gamma after stimulation with PMA/ionomycin. They posited that in these tumors, elevated expression of PD-1 was representative of normal T-cell activation in response to the tumor [46]. As such, although the findings by Coy et al. [33] of elevated PD-1/PD-L1 expression in ACP are exciting and may result in an alternative treatment strategy in resistant and multiply recurrent cases, further investigation is necessary to fully elucidate the implications of this increased PD-1/PD-L1 expression in ACP before any widespread implementation.

### 1.5. CTLA-4 Inhibition and Its Potential Use in the Treatment of ACP

The other major group of immune checkpoint inhibitors that have become increasingly utilized in cancer are the CTLA-4 inhibitors of which ipilimumab is the classic example. One of the first major studies to demonstrate the efficacy of these agents was that by Hodi et al. [47] in 2010. In their study they randomized 676 patients with stage 3 or 4 treatment-resistant melanoma to treatment with ipilimumab plus glycoprotein 100 (gp100) or gp100 alone. They demonstrated a statistically significant, although modest benefit, in terms of survival for patients in the ipilimumab group. Subsequent work by Ji et al. aimed to elucidate what specific factors might be predictive of response to treatment with CTLA-4 blockade [48]. This group utilized gene expression profiling to demonstrate that in pre-treatment samples of patients with metastatic melanoma a higher baseline expression of immune related genes was predictive of an increased response to treatment with ipilimumab [48]. Specifically, they analyzed the gene expression in tumor samples from 45 patients with melanoma both before, and three weeks after treatment with ipilimumab. They found that tumors that had increased expression of immune related genes pre-treatment, were more likely to respond to the therapy. Indeed, when they clustered genes based on biological functions and examined the differential expression of these groups of genes between responders and non-responders, they demonstrated that genes related to the inflammatory response were those that were most differentially expressed between the two groups [48]. This led their group to conclude that a “pre-existing immune-active tumor microenvironment might favor clinical response to ipilimumab”. As previously stated, ACPs have been shown to harbor a significant inflammatory/immune component in both the solid and cystic component and there is mounting evidence that this pro-inflammatory environment plays an active role in tumorigenesis [31]. A 2017 study by Donson et al. [31] utilized various methods to demonstrate upregulation of several pro-inflammatory genes in both the solid and cystic component of these tumors. This begs the question, could the use of a CTLA-4 inhibitor such as ipilimumab lead to improved outcomes in ACP? Furthermore, recent trials have demonstrated that combining different types of immune checkpoint inhibitors can lead to a survival advantage for patients with treatment refractory cancers. Specifically, combining ant-PD1 and anti-CTLA-4 therapy can result in improved survival in treatment-resistant metastatic melanoma, renal cell carcinoma and non-small cell lung cancer [49,50,51,52]. Given the expression of PD-L1 and the significant immune cell and inflammatory milieu present in ACP, the use of such combinations in the treatment of this disease would appear promising. As a result, a lot of work remains to be done to fully elucidate the potential of such treatments in ACP. Given the often-aggressive clinical course, and devastating effects this disease can have on patient’s quality of life such potential is surely worth investigating.

### 1.6. The Role of Senescence and the Senescence Associated Secretory Phenotype (SASP) in the Pathogenesis of ACP

In the normal physiological state, cellular senescence develops in response to both extracellular and intracellular stressors and pushes the cell into cell cycle arrest. This prevents propagation of the damaged cell and, when this occurs in the context of cancer, can ultimately result in tumor suppression [53]. Paradoxically, senescent cells can go on to develop secretory functions that result in changes to the cellular microenvironment and may ultimately promote tumor growth [53]. Senescent cells can persist in a metabolically active state, ultimately developing what is termed the Senescence Associated Secretory Phenotype (SASP) [53]. In such a state, cells can secrete a variety of interleukins, inflammatory cytokines, growth factors, and proteases, which can affect the surrounding cells and tumor microenvironment. SASP factors include pro-inflammatory mediators such as IL-6, IL-1, certain Matrix Metalloproteinases, and various chemokines [53,54]. Of these, Rodier et al. [54] found that IL-6 was the most important in allowing senescent cells to promote cell invasion. Gonzalez-Meljem et al. [55] demonstrated that the SASP plays a prominent role in both genetically engineered mouse models of ACP and human ACP. They used gene set enrichment analysis (GSEA) to demonstrate that beta-catenin accumulating cluster cells in the mouse models of ACP had gene expression profiles that were significantly enriched for SASP genes. Similarly, they utilized ELISA cytokine arrays to demonstrate that multiple SASP associated proteins such as IL-6, IL-1a, MMP2, MMP3, CXCL1, and CXCL11 were all upregulated in the murine cluster cells [55]. In addition, other studies, such as those by Gump et al. [11], and Apps et al. [10], used various techniques to demonstrate the overexpression of several of these proteins in human ACP. Meljem et al. [55] then used laser-capture microdissection and RNA sequencing to analyze the molecular signature of the beta-catenin accumulating cell clusters in human ACP. They performed hierarchical clustering analysis that demonstrated similar molecular profiles between the cluster cells from the mouse models and those from human ACP. Subsequent GSEA of human clusters also demonstrated a strong SASP signature. They thus concluded that the human and mouse clusters represent equivalent structures and share a common senescent molecular signature. Given the critical role that these cell clusters are thought to play in ACP tumorigenesis, they posited that the SASP may play a critical role in the pathogenesis of ACP [55]. This paper aimed to demonstrate the critical role played by inflammation in the pathogenesis of ACP. Given that human ACPs seem to harbor a very strong SASP signature and the SASP induces a strong pro-inflammatory state, it is very possible that the SASP plays a critical role in producing the pro-inflammatory milieu and invasive nature of ACP. Trials examining the use of senolytic drugs are currently in their incipient stages and it is possible that such therapies may provide an attractive treatment strategy for ACP in the future [56].

## 2. Conclusions

A significant and growing body of evidence points to a critical role in the activation of inflammation and the immune response in the pathogenesis of ACP. Multiple studies have demonstrated high levels of inflammatory markers and cytokines in both ACP cyst fluid and solid tumor. Many of these employed advanced genomic, proteomic and transcriptomic techniques to demonstrate expression of multiple genes involved in the inflammatory and immune response in these tumors. A number of these markers represent attractive potential targets for directed therapy in the treatment of ACP. Specifically, due to existing experience combined with proven efficacy in other cancers and diseases, IL-6 and the immune checkpoint inhibitors (anti-PD-1/PD-L1 and anti-CTLA-4) may represent particularly good targets/therapies. Similarly, combinations of such agents have proven very effective in prolonging survival in malignant cancer such as melanoma, renal cell carcinoma and non-small-cell lung cancer that were failing more traditional treatment. Such combination therapy may also present a potential therapeutic strategy in the management of recurrent and treatment-resistant ACP. In addition, these agents might also be combined with agents that do not specifically interact with inflammatory/immune processes (e.g., MEK inhibition). In fact, Apps et al. [10] demonstrated that MAPK/ERK pathway likely plays a pivotal role in the pathogenesis of both murine and human ACP. In addition, they showed that treating human ACP with trametinib ex vivo resulted in decreased proliferation and increased apoptosis. Finally, recent work has also demonstrated the pivotal role played by senescence and the SASP in the pathogenesis of these tumors. It is possible that the strong SASP signature drives much of the inflammation seen in ACP, and that targeting SASP associated pathways may provide an effective treatment strategy in the future. Due to the benign histological nature of the disease, it is likely that initial clinical trials of such agents will be reserved for patients with recurrent or progressive disease. In addition, due to the rarity of the disease and the scarcity of tumor tissue it is vital that pediatric centers continue to work together to share knowledge and tissue in an effort to accelerate the development of safe and efficacious treatments. Such efforts will hopefully result in improved outcomes for children suffering from this chronic and often devastating disease. Finally, other advanced techniques are being developed that continue to enhance our ability to better diagnose, and identify biomarkers in oncologic diseases that may result in the development of better therapeutics [57,58]. It is possible that such techniques if applied to ACPs could result in significant advances in the diagnosis and treatment of ACP in the future.

## Figures and Tables

**Table 1:** Key studies that have demonstrated the key role of the inflammatory/immune response in the pathogenesis of adamantinomatous craniopharyngioma.

Study	Summary of Study	Findings
Kilday et al. 2017 [35]	Multinational study assessing the efficacy of intra-cystic IFN-alpha in treating ACP	Demonstrated a progression free survival advantage for intracystic IFN-alpha
Pettorini et al. 2010 [36]	Identified the presence of alpha-defensins 1–3 in ACP cyst fluid	Demonstrated the importance of inflammation the genesis of ACP cysts
Gump et al. 2015 [11]	Used mRNA microarray analysis to identify the overexpression of multiple inflammatory markers in ACP relative to other tumors	Identifies IL6R and IL2RB to be overexpressed in ACP relative to normal brain and other tumors
Donson et al. 2017 [31]	Identified elevated levels of severeal inflammatory markers in both ACP cyst fluid and solid tumor	Overexpressed inflammatory markers identified included IL-6, IL-8, CXCL1, and IL-10
Apps et al. 2018 [10]	Used various methods including RNA sequencing to identify activation of the inflammasome in ACP cyst fluid and solid tumor	Imflamatory genes that were overexpressed included IL-1B, IL-18, IL-6, IL-8, IL-10
Coy et al. 2018 [33]	demonstrated the expression of PD-L1 in epithelial cells lining the cysts and intrinsic PD-1 expression in the beta-catenin over expressing whorl-like epithelial cell clusters in ACP	The first paper to demonstrate that immune checkpoint inhibitors may play a role in ACP treatments
